# Supplementary material for: MetaRibo-Seq measures translation in microbiomes
Source: Nat Commun. 2020 Jun 29;11:3268. doi: 10.1038/s41467-020-17081-z (PMC7324362; doi:10.1038/s41467-020-17081-z)
Supplement: Supplementary file 10 — Supplementary Data 7 [file 41467_2020_17081_MOESM10_ESM.zip › File2/Confidence_VeryHigh_Taxonomy/221470_out.krona.html]

Javascript must be enabled to view this page.

members
magnitude
magnitudeUnassigned
count
unassigned
taxon
rank

221470\_out

39

39
2
superkingdom

1239
38
phylum

class
38
186801

38
186802
order

genus
2039302
1


SRS053335\_contig\_number\_15921
1981510
1
species

family
37
31979

genus
2

SRS024435\_contig\_number\_contig-100\_29514.29514SRS104311\_contig\_number\_20213
1485
37


SRS011134\_contig\_number\_contig-100\_6617.114283SRS012273\_contig\_number\_13844SRS013951\_contig\_number\_contig-100\_3199.217024SRS013965\_contig\_number\_36128SRS014235\_contig\_number\_8330SRS015190\_contig\_number\_contig-100\_21181.62809SRS019267\_contig\_number\_contig-100\_1274.1274SRS022137\_contig\_number\_12872SRS024435\_contig\_number\_contig-100\_904.904SRS042628\_contig\_number\_contig-100\_3500.109497SRS043001\_contig\_number\_contig-100\_18811.18811SRS043411\_contig\_number\_3845SRS043768\_contig\_number\_16240SRS063985\_contig\_number\_14424SRS077335\_contig\_number\_contig-100\_121.43236SRS104311\_contig\_number\_39363SRS148159\_contig\_number\_contig-100\_19849.19850
1262789
17
species

species
10
1262799

SRS015960\_contig\_number\_4859SRS019397\_contig\_number\_4892SRS023583\_contig\_number\_contig-100\_23572.57898SRS043001\_contig\_number\_contig-100\_966.62954SRS045244\_contig\_number\_7231SRS045826\_contig\_number\_15866SRS054905\_contig\_number\_20631SRS075984\_contig\_number\_24136SRS101433\_contig\_number\_contig-100\_31274.71937SRS147557\_contig\_number\_19489


SRS047044\_contig\_number\_3590SRS147766\_contig\_number\_contig-100\_6947.106487
1262807
2
species


SRS012273\_contig\_number\_1738SRS014948\_contig\_number\_17630SRS022071\_contig\_number\_36370SRS047433\_contig\_number\_12182SRS062427\_contig\_number\_9990SRS148970\_contig\_number\_4299
1896989
6
species

1
976
phylum

class
200643
1

order
171549
1

family
815
1

1
816
genus

47678
1
species

SRS056259\_contig\_number\_737
